# Treatment with Actovegin improves spatial learning and memory in rats following transient forebrain ischaemia

**DOI:** 10.1111/jcmm.12297

**Published:** 2014-05-06

**Authors:** Sigal Meilin, Fausto Machicao, Martin Elmlinger

**Affiliations:** a Neurology Service, MD Biosciences LtdNes-Ziona, Israel; b Molecular Genetics and Diagnosis, Department of Internal Medicine IV, University HospitalTübingen, Germany; c Experimental Medicine, Biomarker Development, Takeda Pharmaceuticals International GmbHZurich, Switzerland

**Keywords:** actovegin, ischaemia, learning, memory, neuroprotection, stroke

## Abstract

This study aimed to investigate whether Actovegin, which is a deproteinized ultrafiltrate derived from calf blood, demonstrates neuroprotective effects in a rat model of transient global cerebral ischaemia. Forty Sprague Dawley rats were subjected to four-vessel occlusion to induce transient global cerebral ischaemia followed by either saline or Actovegin treatment. Sham operations were performed on 15 rats. Actovegin (200 mg/kg) or saline was administered 6 hrs after carotid artery occlusion and then daily until Day 40. Learning and memory were evaluated using the Morris water maze test over two different 5-day periods, and grip strength testing was also performed to control for potential motor impairments. Rat brains were harvested for histological analysis on Day 68. In comparison to controls, Actovegin-treated rats exhibited a decreased latency to reach the hidden platform on the second learning trial of water maze testing (46.82 ± 6.18 *versus* 27.64 ± 4.53 sec., *P* < 0.05; 38.3 ± 8.23 *versus* 13.37 ± 2.73 sec., *P* < 0.01 for the first and second 5-day testing periods, respectively). In addition, Actovegin-treated rats spent more time in the platform quadrant than saline-treated rats during memory trials (*P* < 0.05). No differences in grip strength were detected. Histological analyses demonstrated increased cell survival in the CA1 region of the hippocampus following Actovegin treatment (left hemisphere, 166 ± 50 *versus* 332 ± 27 cells, *P* < 0.05; right hemisphere, 170 ± 45 *versus* 307 ± 28 cells, *P* < 0.05, in saline- *versus* Actovegin-treated rats, respectively). In rats, Actovegin treatment improves spatial learning and memory following cerebral ischaemia, which may be related to hippocampal CA1 neuroprotection.

## Introduction

Ischaemic stroke is an increasing clinical and economic burden worldwide [1,2]. Long-term disabilities, such as post-stroke cognitive impairment (PSCI), frequently occur following a stroke [3], and up to 22% of all stroke patients may remain clinically demented 3 months after the initial ischaemic insult [4]. Thus, the continued treatment of ischaemic stroke and its sequelae urgently warrants additional studies, particularly because the World Health Organization projects that cerebrovascular disease will continue to be one of the leading causes of mortality in the near future [5].

Recent developments in our understanding of the pathophysiology of ischaemia-induced neuronal damage have stimulated an interest in new neuroprotective therapies [6], which may potentially protect against stroke sequelae, including PSCI. Various therapies have been evaluated [7], and although many of these therapies have failed in clinical trials [7,8], thrombolysis trials have demonstrated the existence of a salvageable penumbra following arterial occlusion [9]. This observation has revitalized therapeutic neuroprotection research for stroke therapies.

The pathophysiology of stroke is complex, but the inhibition of any one of the multiple molecular events with therapeutic agents may partially protect brain tissue from injury [10]. For example, increasing evidence suggests that oxidative stress significantly contributes to the observed neuronal cell death following ischaemic stroke. Reperfusion of ischaemic tissue has been suggested to cause the release of reactive oxygen species (ROS) and reactive nitrogen species from mitochondria [11], which results in blood–brain barrier dysfunction and other detrimental effects in the surrounding tissues [12,13].

Actovegin, which is a drug based on a deproteinized ultrafiltrate derived from calf blood (≤5000 D) [14], is one treatment currently under investigation in a good clinical practice-compliant, double-blind, *placebo*-controlled trial for the treatment of PSCI (ARTEMIDA; http://clinicaltrials.gov identifier: NCT01582854). Clinical trials in other patient populations have demonstrated that Actovegin improves clinical outcome in both diabetic polyneuropathy (DPN) [15] and mixed dementia patients [16,17], although its effects on acute cerebral ischaemia have not been thoroughly investigated. *In vitro* and *in vivo* assays investigating the mode of action of Actovegin have revealed pleiotropic neuroprotective and metabolic effects.

Together with these findings, experiments using streptozotocin (STZ)-treated rats exhibiting severe neuropathic symptoms have demonstrated that Actovegin can reduce the degeneration of peripheral neurons and improve their functionality. Changes in poly (ADP-ribose) polymerase (PARP) activity have been observed in rat neurons treated with Actovegin and have been implicated in its mechanism of action [18]. Furthermore, *in vitro* experiments using primary hippocampal neuron cultures have demonstrated that Actovegin can reduce oxidative stress and apoptosis, thereby increasing cell survival [19]. The observed reduction in apoptosis has been suggested to involve the modulation of the nuclear factor kappa B (NF-κB) pathway [20]. Other studies have demonstrated that Actovegin enhances glucose uptake, energy metabolism, and oxygen uptake and utilization and that it accelerates wound healing at the molecular level [21]. However, the cellular and molecular effects of Actovegin treatment following ischaemia have not yet been characterized, as studies to date have focused on *in vitro* models and other disease models, including diabetic polyneuropathy.

With this in mind, the objective of this study was to address whether Actovegin confers neuroprotection in an established rat model of global cerebral ischaemia, and if so, whether this effect is related to improvements in cognitive and neurological performance. To assess the effects of Actovegin following cerebral ischaemia, the four-vessel occlusion (4-VO)-induced stroke model was chosen as the model system for this study because it is a reliable and widely used rat model of transient global forebrain ischaemia.

## Materials and methods

### Animals

Fifty-five male Sprague Dawley rats (which have been used in similar studies [22,23]) weighing ∼250 g (±20%) were included in this study. Rats were allowed a minimum of 5 days to acclimate to their surroundings and were provided food (a commercial, sterile rodent diet) and water *ad libitum*. The study was approved by the Committee for Ethical Conduct in the Care and Use of Laboratory Animals. All attempts were made to maximize animal welfare while preserving scientific validity and integrity.

### The 4-VO-induced stroke model

The 4-VO model was selected because it results in hippocampal injury and a subsequent decline in cognitive performance [24–26]. Once the rats were fully anaesthetized with ketamine (90 mg/kg) and xylazine (10 mg/kg), the first cervical vertebrae of the rats were exposed, and the vertebral arteries were permanently occluded by electrocauterization. The common carotid arteries were then isolated through a ventral midline neck incision and lifted using a 4-0 silk ligature. Twenty-four hours after this initial procedure, the rats were reanaesthetized with isoflurane. Subsequently, the common carotid arteries were exposed and occluded for 15 min. using micro aneurysm clips [22]. A period of 20 min. of forebrain ischaemia was also considered, but this occlusion period has been associated with a high rate of mortality [27]. During this procedure, global ischaemia was verified by a transient cessation of anaesthesia during carotid artery occlusion. Rats lacking a righting reflex were considered ischaemic and were included in this study [22]. After removal of the micro aneurysm clips, the skin at the incision site was sutured, and the rats were returned to their cages to recover under heating lamps. Sham-operated animals were subjected to identical operational procedures but were not subjected to vascular occlusion.

During the procedure, the core temperature of each animal was monitored using a rectal probe (Model 400; YSI Inc., Yellow Springs, OH, USA) connected to a thermometer (Model 8402-00; Cole-Parmer Instrument Co. Ltd, London, UK). The ischaemic insult was initiated when a rectal temperature of 37–38°C was achieved, and this temperature was maintained throughout the procedure.

**Fig. 1:**
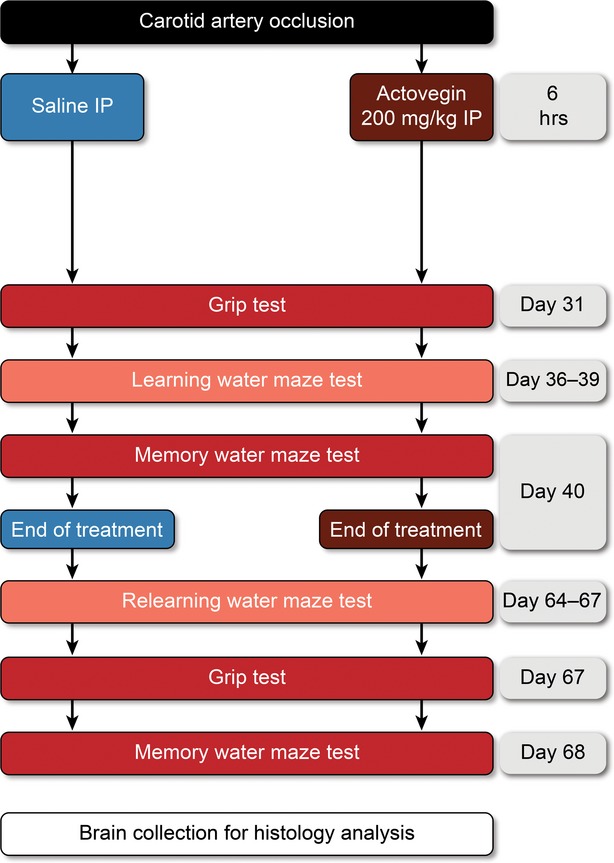
Study experimental design. Actovegin (200 mg/kg) and saline were administered 6 hrs after carotid artery occlusion until Day 40. Grip testing was performed on Days 31 and 67; learning water maze trials were performed on Days 36–39 and 64–67; and memory water maze trials were performed on Days 40 and 68.

Following surgery, the rats were randomly assigned to one of three experimental groups: sham-operated, saline treatment or Actovegin treatment. Actovegin (200 mg/kg) and saline were intraperitoneally (IP) administered 6 hrs after carotid artery occlusion and once daily until Day 40 (Fig. 1). This dose of Actovegin has been previously calculated to be equivalent to the dose used in human DPN patients, and daily Actovegin infusions are frequently prescribed in clinical practice. In addition, this dosing regimen has been previously used in neuropathic rat studies [18].

### Morris water maze

The Morris water maze was selected because it is one of the most common functional methods used to assess hippocampal injury. Two periods of Morris water maze tests were completed during the study (Fig. 1; Days 36–40 and 64–68 after common carotid artery occlusion) to evaluate the effects of Actovegin on learning and memory. These tests were performed according to a modified protocol that has been previously described by Raz *et al*. [28], and Actovegin-treated rats were compared with saline-treated and sham-operated rats. The selected time-points assessed the effects of Actovegin in the stationary phase of neuronal injury [29] rather than during levels of high inflammatory activity [30].

Rats were introduced to a standardized 1.2 m-diameter pool filled with water for 120 sec. or until they located a platform hidden 1 cm below the water surface. Several visual cues were provided within the room in which the pool was located to allow rats to spatially navigate the water maze. Specifically, one tube was positioned from east to west on the north wall, an ‘X’ was marked on the south wall, a second tube was positioned from the ceiling to the floor on the west wall, and the entrance door was located on the east wall. Rats that located the hidden platform were allowed to remain on it for 10 sec., and rats that failed to find the platform within 120 sec. were placed on the platform for 10 sec. Rats were allowed two attempts to find the hidden platform, and this *learning* test was performed over a period of 4 days (Days 36–39 and Days 64–67; Fig. 1). *Memory* tests (probe trials) were performed on the fifth day (Days 40 and 68), at which time the hidden platform was removed, and rats were placed in the pool for a single 60-sec. trial. The amount of time that each rat spent in the quadrant where the hidden platform had previously been located was recorded by an observer who was blinded to the experimental groups.

The Morris water maze was used to evaluate how well the rats remembered the location of the hidden platform and whether they had learned to navigate towards the appropriate quadrant. The second series of Morris water maze tests (Days 64–68) was conducted to evaluate whether the effects of Actovegin persist following treatment cessation, and therefore whether it may have disease-modifying properties.

### Grip testing

As a control, grip testing was performed to objectively quantify rat muscular strength. A Grip Strength Meter (Cat #47200; Ugo Basile Srl, Monvalle Varese, Italy) measured the forelimb grip strength for each rat twice on Day 2 to obtain a baseline value and again on Days 31 and 67 (Fig. 1). Briefly, the rats were gently held by their tails before being placed over the top of a grid such that their front paws could grip the strength meter. Rats were then pulled backwards until they could no longer hold onto the meter, thereby measuring their maximal grip strength.

### Histological analysis

Neuronal damage inflicted by the 4-VO model can be assessed using quantitative histological measurements. In the present study, hippocampal cornu ammonis 1 (CA1) pyramidal cell survival was determined. At the end of the study (Day 68), rats were killed with pentobarbital sodium (>100 mg/kg IP) and were then perfused with saline and 4% formaldehyde through the left ventricle. The brains were post-fixed in formaldehyde for at least an additional 72 hrs. Rat brain blocks were prepared using standard paraffin-embedding techniques. After tissue embedding, 5-μm hippocampal sections were cut, stained with haematoxylin and eosin, and examined through a standard analysis of surviving (*i.e*. viable) cells [31] by an observer who was blinded to the experimental conditions. For quantitative histological analyses of the hippocampal CA1 region, the region was divided into three areas. In each area, CA1 neurons that appeared normal were counted, and the sum of all three areas represented the total number of normal CA1 neurons per region.

Following this analysis, all paraffin-embedded blocks were stored until subsequent analysis.

### Statistical analysis

The data were analysed using Student's *t*-test (Prism V 4.0; GraphPad Software Inc., La Jolla, CA, USA) and one-way anova followed by Tukey's multicomparison analysis. *P* < 0.05 was considered significant.

## Results

### Cell survival

**Fig. 2:**
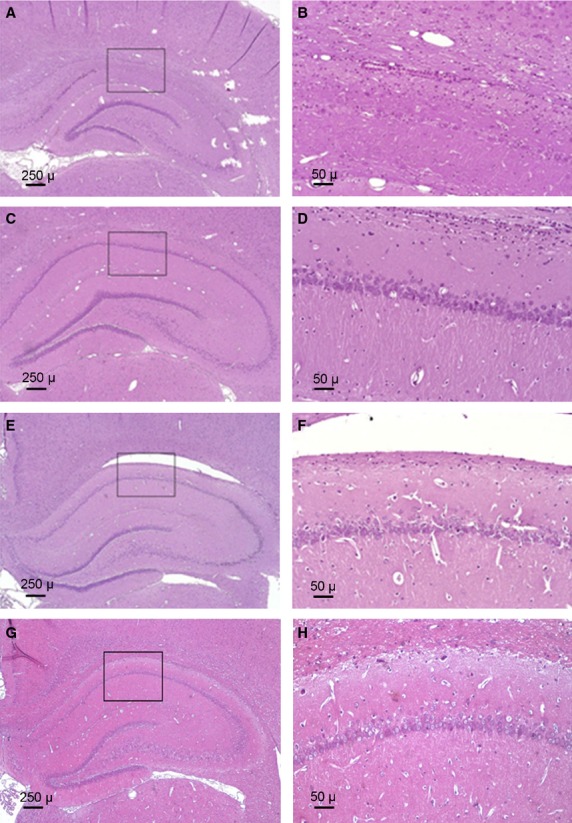
Photomicrographs of paraffin-embedded brain sections stained with haematoxylin and eosin. The boxed CA1 subfield sections are shown at higher magnification (**B**, **D**, **F** and **H**), and the number of viable neurons in these areas was counted. (**A** and **B**) demonstrate a nearly complete loss of CA1 neurons in a saline-treated control rat. (**C** and **D**) depict the nearly complete preservation of CA1 neurons in an Actovegin-treated rat. (**E** and **F**) demonstrate partial preservation of CA1 neurons in an Actovegin-treated rat. (**G** and **H**) show intact CA1 neurons in a sham-operated rat.

**Fig. 3:**
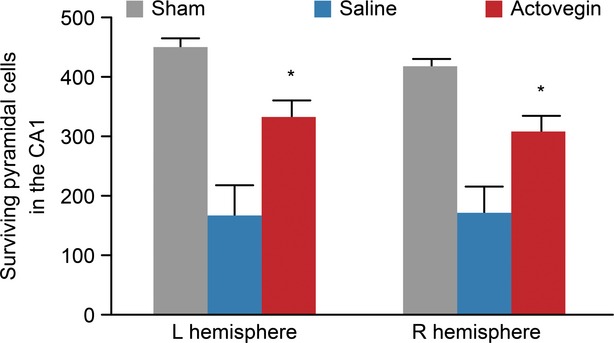
Hippocampal CA1 pyramidal cell survival. The data are presented as the means ± the standard errors of the mean (*P < 0.05 *versus* saline).

Global cerebral ischaemia resulted in a significant reduction in the number of viable (*i.e*. surviving) hippocampal CA1 pyramidal cells. Histological analyses (Fig. 2) indicated that Actovegin treatment protected against CA1 cell death. In comparison to controls, Actovegin treatment significantly increased the mean number of viable cells (left hemisphere, 332 ± 27 *versus* 166 ± 50 cells in Actovegin- *versus* saline-treated rats, respectively, *P* < 0.05; right hemisphere, 307 ± 28 *versus* 170 ± 45 cells in Actovegin- *versus* saline-treated rats, respectively, *P* < 0.05; Fig. 3). The degree of neuroprotection between individual animals was variable but remained significant between the groups.

### Learning and memory

**Fig. 4:**
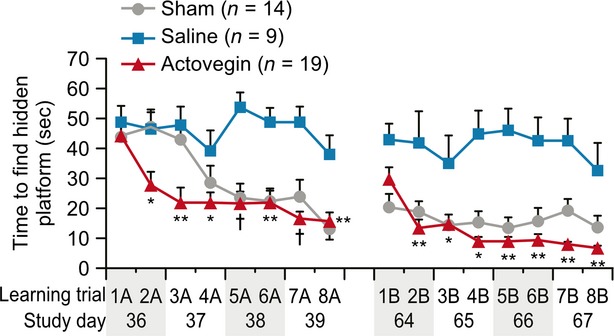
Learning Morris water maze trials (Days 36–39 and 64–67 following common carotid artery occlusion). Data points represent the means ± the standard errors of the mean (*P < 0.05; **P < 0.01; †*P* < 0.001 Actovegin *versus* saline).

**Fig. 5:**
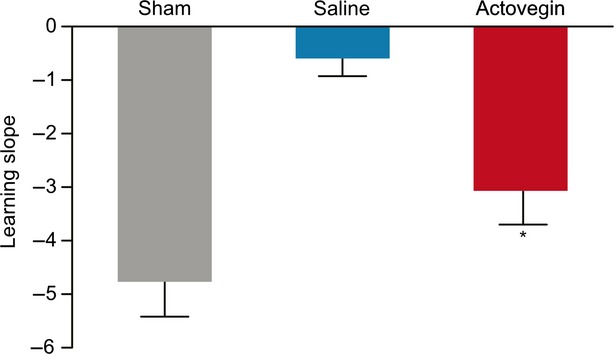
Rat learning slopes calculated from Day 40 Morris water maze testing. Data points represent the means ± the standard errors of the mean (*P = 0.019 *versus placebo*).

The rats treated with Actovegin (*n* = 19) took significantly less time than saline-treated controls (*n* = 9) to find the hidden platform by the second learning trial of the first series of water maze tests (Days 36–39; 27.64 ± 4.53 sec. for the Actovegin group *versus* 46.82 ± 6.18 sec. for the control group, *P* < 0.05). Similar results were observed during the second series of water maze tests (Days 64–67), during which Actovegin-treated rats took significantly less time than controls to find the hidden platform by the second learning trial (13.37 ± 2.73 sec. for the Actovegin group *versus* 38.3 ± 8.23 sec. for the control group, *P* < 0.01). During both series of learning tests, the significant differences between the Actovegin- and saline-treated groups persisted in all subsequent trials (Day 39, 15.75 ± 2.94 and 38.02 ± 6.45 sec. for the Actovegin- and saline-treated groups, respectively, *P* = 0.001; Day 67, 6.76 ± 0.52 and 32.48 ± 9.34 sec. for the Actovegin- and saline-treated groups, respectively, *P* < 0.01; Fig. 4). The learning slopes were calculated at Day 40 and confirmed that Actovegin-treated rats exhibited a significantly increased learning rate in comparison to saline-treated rats (*P* = 0.019; Fig. 5).

**Fig. 6:**
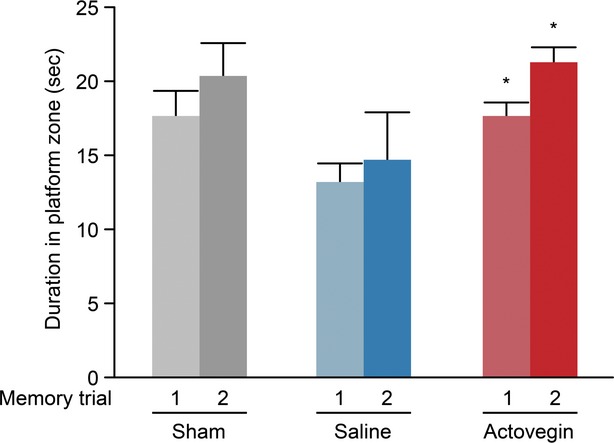
Day 40 (memory test #1) and Day 68 (memory test #2) memory trials obtained from the Morris water maze. Data points represent the mean time that rats spent in the platform quadrant (seconds) ± the standard error of the mean (*P < 0.05 *versus* saline).

Moreover, the memory trials revealed that Actovegin-treated rats spent significantly more time in the platform quadrant than saline-treated rats on Day 40 (17.72 ± 0.84 and 13.17 ± 1.24 sec. for Actovegin- and saline-treated rats, respectively, *P* < 0.05) and Day 68 (21.29 ± 1.00 and 14.70 ± 3.17 sec. for Actovegin- and saline-treated rats, respectively, *P* < 0.05; Fig. 6).

### Muscle strength

**Fig. 7:**
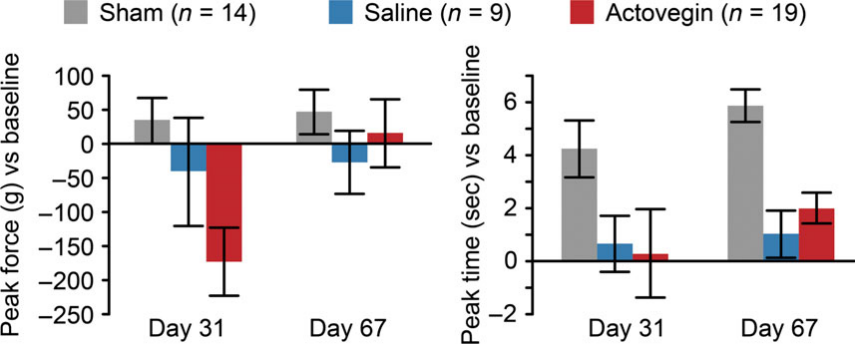
The peak pull force (g) achieved by rat forelimbs (left panel) and peak time required to achieve the maximum muscle strength in the grip test (right panel). Data represent the means ± the standard errors of the mean.

At all time-points examined, Actovegin treatment did not significantly affect muscle strength (*i.e*. the time to achieve maximum gripping force or the peak pull force) in comparison to saline treatment (Fig. 7). A trend towards a reduction in peak pull force was observed in Actovegin-treated animals at Day 31, but this reduction did not achieve significance.

### Body weight

All animals experienced minor weight loss following vertebral artery occlusion, but this weight loss was completely reversed. All animals gained weight during the study, and no significant weight differences were observed between the treatment groups. In addition, all rats were normoglycaemic throughout the study.

### Survival

Three weeks after global cerebral ischaemia, the mortality rate of the saline-treated group was 55% (of the 20 animals that were subjected to 4-VO, eight died within the first week, and three died during the following 2 weeks). In comparison, the mortality rate of Actovegin-treated rats over this period was only 5% (1/20 animals that were subjected to 4-VO). Of the 15 sham-operated animals, no fatalities were observed, but one animal was excluded from the study and culled because it exhibited abnormal behaviour. Only animals that survived the entire study were included in the behavioural analyses.

## Discussion

The neuroprotective effects of Actovegin on peripheral and hippocampal rat neurons have been previously demonstrated *in vivo* and *in vitro*, respectively [18,19]. We conducted the present study to investigate the neuroprotective effects of Actovegin in a 4-VO rat model of transient global cerebral ischaemia.

Rat studies have shown that hippocampal lesions impair recognition memory [32] and the ability to perform object recognition and spatial awareness tasks [33]. Collectively, these results highlight the importance of hippocampal processing for both spatial and recognition memory and emphasize the critical role that CA1 neurons play in the consolidation of these processes.

Although the functional role of CA1 neurons has been defined in rodents, only a limited number of studies have demonstrated functional roles of human and primate CA1 neurons [34,35]. Nevertheless, one study examining transient global amnaesia (TGA) patients found that the disorder significantly affected place learning [35]. These results may be relevant to our study because TGA, similar to the 4-VO-induced stroke model, results in selective acute and focal hippocampal CA1 lesions [24,25].

The cell survival analysis in our study demonstrated that Actovegin treatment significantly decreased hippocampal CA1 cell death in comparison to saline treatment. These results are consistent with the findings of Hoyer and Betz, who previously reported that Actovegin confers cellular protection in a hypoxic model of cerebral ischaemia [36]. Hippocampal CA1 neurons have been shown to be more vulnerable to oxidative stress than cerebral cortical and hippocampal CA3 neurons [37], and the protection of hippocampal CA1 neurons by Actovegin may be partially because of its antioxidative [19] and metabolic effects [38].

We suggest that hippocampal CA1 neuroprotection may be responsible for the observed significant improvement in learning and memory of the Actovegin-treated rats in Morris water maze tests. In support of this hypothesis, we observed nearly twice as many surviving hippocampal CA1 neurons in Actovegin-treated rat brains in comparison to saline-treated rat brains at the end of the study.

Derev'yannykh *et al*. have conducted a preliminary study of short-term treatment with Actovegin in 32 post-stroke patients and found that patients exhibited improved short-term memory, speech function and concentration [39]. In our experimental study, saline-treated rats required nearly three times as long as Actovegin-treated rats to locate the hidden platform during the second period of water maze learning trials. Moreover, significant differences in learning and memory between the two groups persisted even after treatment cessation, suggesting that Actovegin may possess a disease-modifying mode of action. Therefore, these results provide the first *in vivo* mechanistic evidence for the protection of cognitive function in global cerebral ischaemia by Actovegin and support the rationale of the ongoing ARTEMIDA trial (http://clinicaltrials.gov identifier: NCT01582854), which aims to investigate the efficacy and safety of Actovegin in PSCI.

The results of the grip test did not reveal any significant differences between saline- and Actovegin-treated rats, suggesting that muscle weakness did not contribute to the Morris water maze results. In addition, weight loss reversed within 1 week of vertebral artery occlusion, indicating that all rats were generally in good health throughout the study and were able to access food and water.

### Study limitations

Despite the clinical relevance of this *in vivo* study, limitations associated with the experimental approach should be considered. We observed that cerebral ischaemia significantly affected the ability of rats to perform a spatial learning and memory task, a finding that has been confirmed in another study using the 4-VO model in middle-aged rats [40]. However, we cannot exclude whether a lack of motivation (*i.e*. the translation of knowledge into action), rather than cognitive deficits, contributed to this result. Moreover, baseline learning and memory were not assessed, which makes it difficult to determine whether pre-existing performance discrepancies contributed to the differences between the groups. The Morris water maze test also requires complex and coordinated movements and is sensitive to certain aspects of learning and memory. Although movement and coordination are often impaired in stroke, the Morris water maze may not be the most clinically meaningful experimental test for stroke. Thus, other tests, such as radial arm mazes and operant delayed matching tasks that measure additional aspects of learning and memory would be helpful to confirm our findings.

Similarly, hippocampal lesions have been shown to impair spatial awareness [33], but surgery or ischaemia in this study may also have unintentionally caused deficits in the brain areas that are responsible for other behaviours. Furthermore, the performance of the animals may have been affected by individual differences in visual acuity or swimming ability. An additional assessment of muscle strength and endurance, such as a swim speed test, would be useful to confirm that surgery did not cause excessive muscle trauma and that reduced motor function was not a contributing factor to the observed learning and memory impairments. Additional experiments designed to examine these questions would help to confirm the findings of the present study.

## Conclusions

Our results demonstrate that administering Actovegin 6 hrs after global cerebral ischaemia and once daily for 40 days results in neuroprotection, and significantly improves spatial learning and memory in Sprague Dawley rats. We postulate that the mechanism of this improved cognitive performance relies on the neuroprotective action exerted by Actovegin in the CA1 region of the rat hippocampus. Successful translation of these results to humans may significantly aid in the management of stroke and its debilitating consequences.
